# Single-molecule RNA capture-assisted droplet digital loop-mediated isothermal amplification for ultrasensitive and rapid detection of infectious pathogens

**DOI:** 10.1038/s41378-023-00576-2

**Published:** 2023-09-25

**Authors:** Liying Jiang, Xianghao Lan, Linjiao Ren, Zhiyuan Jin, Xuchen Shan, Mingzhu Yang, Lingqian Chang

**Affiliations:** 1 School of Electrical and Information Engineering, Zhengzhou University of Light Industry, 450002 Zhengzhou, China; 2 Academy for Quantum Science and Technology, Zhengzhou University of Light Industry, 450002 Zhengzhou, China; 3 Beijing Advanced Innovation Center for Biomedical Engineering, School of Biological Science and Medical Engineering, Beihang University, 100191 Beijing, China; 4 School of Physics, Beihang University, 100191 Beijing, China; 5 Beijing Research Institute of Mechanical Equipment, 100143 Beijing, China

**Keywords:** Microfluidics, Biosensors

## Abstract

To minimize and control the transmission of infectious diseases, a sensitive, accurate, rapid, and robust assay strategy for application on-site screening is critical. Here, we report single-molecule RNA capture-assisted digital RT-LAMP (SCADL) for point-of-care testing of infectious diseases. Target RNA was captured and enriched by specific capture probes and oligonucleotide probes conjugated to magnetic beads, replacing laborious RNA extraction. Droplet generation, amplification, and the recording of results are all integrated on a microfluidic chip. In assaying commercial standard samples, quantitative results precisely corresponded to the actual concentration of samples. This method provides a limit of detection of 10 copies mL^−1^ for the N gene within 1 h, greatly reducing the need for skilled personnel and precision instruments. The ultrasensitivity, specificity, portability, rapidity and user-friendliness make SCADL a competitive candidate for the on-site screening of infectious diseases.

## Introduction

Infectious pathogens (bacteria, viruses, parasites, etc.) affect humans and animals, posing acute threats to public health^1,2^. Prevention and control of infectious diseases are key to minimizing pathogen damage, and the mass screening of high-risk populations, early identification of infected cases and asymptomatic infections, and breaking the chain of transmission are effective strategies to avoid rapid geographic spread of infectious disease^3,4^. However, for diseases with a high transmissibility rate and low infectious dose, such as COVID-19, it is a challenge to rapidly and accurately diagnose infected cases, especially asymptomatic and presymptomatic carriers^5,6^. Approaches with ultrasensitivity, high specificity and rapidity, as well as user-friendliness and simplicity, are promising alternatives for mass screening to reduce disease transmission of pandemic viruses^7–9^.

Due to its high sensitivity and specificity, real-time quantitative polymerase chain reaction (real-time qPCR) is the gold standard for detecting viruses from infectious samples in most clinical laboratories^10,11^. However, it is still difficult to avoid misdiagnosis by real-time qPCR when handling specimens with low viral loads^12^. Misdiagnosis is commonly caused by limited sensitivity and reduced concentration after elution in extraction procedures^13^. In extraction based on commercial kits and platforms, several hundred microliters or even smaller volumes of samples are processed, and only a minority of the extracted nucleic acid (usually <20 μL) ultimately participates in amplification^14^. Moreover, the need for skilled personnel and sophisticated thermal cycling instruments limits the application of qPCR in laboratories with poor operational conditions in resource-limited areas^9,15,16^. Although alternative isothermal amplification approaches such as recombinase polymerase amplification (RPA), loop-mediated isothermal amplification (LAMP) and CRISPR-based techniques have been developed to accommodate point-of-care (POC) applications without requiring bulky instruments and time-consuming steps^17–20^, the majority of them still rely on laborious nucleic acid extraction, which limits their use in laboratory settings^5,21–23^. Although strategies that can detect RNA without extraction steps have been developed, they sacrifice high sensitivity or specificity^24^. In recent years, droplet microfluidic technology has been widely used in biomolecule detection. The local concentration of the detection target can be improved by droplet microfluidics. Compared with traditional nucleic acid detection methods, its sensitivity is more than 10000 times higher, and absolute quantification of the target molecule is realized^25^. Thus, droplet amplification is beneficial to improve the sensitivity and accuracy of detection.

In this study, we developed single-molecule RNA capture-assisted digital RT-LAMP (SCADL) for the ultrasensitive, specific and rapid detection of infectious viruses. To eliminate the cost-, time-, labor-, and reagent-consuming RNA extraction step, a group of capture probes (CPs) was designed for the capture and enrichment of the target sequences. A microfluidic chip that integrates the generation of droplets, digital RT-LAMP of target RNA, and analysis of results is designed and fabricated. We validated the performance of SCADL with the detection of SARS-CoV-2, achieving a limit of detection (LOD) of 10 copies mL^−1^, with a detection duration within 1 h. More importantly, the process of SCADL only includes several simple operations. The rapid, sensitive, specific, and user-friendly approach is a promising candidate for on-site screening of infectious pathogens in both infected carriers and environments.

## Results

**Fig. 1:**
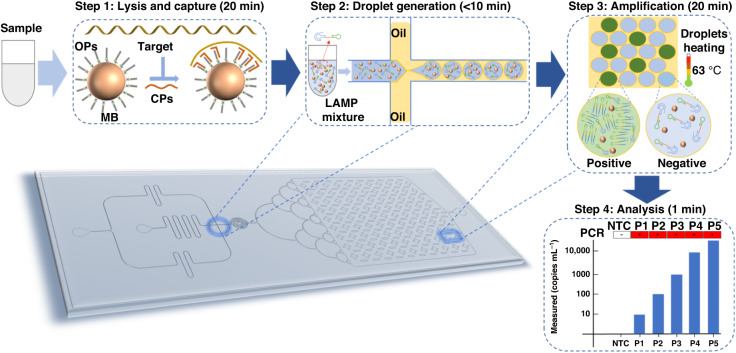
Schematic illustration of the SCADL strategy. Steps for SCADL include lysis and capture (20 min), droplet generation (10 min), RT-LAMP (20 min) and analysis (1 min). Target sequences are captured and enriched by specific capture probes (CPs) bonded on the surface of magnetic beads (MBs) through hybridization with the oligonucleotide probes (OPs) modified on it. The complex of target sequences and MBs, together with the mixture for RT-LAMP, are dispersed into over 2 × 10^4^ droplets. A droplet with at least one copy of the target can generate amplification and yield fluorescent signals at end-point analysis. Quantitative analysis of the target can be completed within 1 min by calculating the number of droplets with fluorescent signals

We chose the N gene of SARS-CoV-2 as the target for validation, while all the probes and primers were designed according to the sequence of the N gene. The whole assay of SCADL includes two steps: lysis and capture in tubes (20 min) and droplet generation and amplification on chips (30 min), in which CPs specifically capture the target RNA instead of RNA extraction (Fig. 1). All the capture probes contain a universal “tail”, enabling them to bond to the surface of MBs through hybridization between the “tail” and the oligonucleotide probes (OPs) modified on the surface of MBs. Benefiting from the base-stacking effect of all CPs, the capture of target RNA is effective and efficient. Furthermore, magnetic separation of MBs makes the system capable of handling up to 1 mL of sample or even greater volume. Thus, sensitivity can be improved by merely increasing the volume of samples. All the targets enriched through capture by MBs, together with the mixture for RT-LAMP, are subsequently separated into approximately 2 × 10^4^ droplets and finally amplified by RT-LAMP, in which each droplet has at most 1–2 copies of target. Amplification based on droplets further improves the sensitivity and achieves absolute quantification of the targets. On the microfluidic chip, droplets are generated through specially designed channels (crossing microchannel) within 10 min. Then, all the droplets spread into the collection chamber and form a monolayer of droplets for amplification and signal recording. In RT-LAMP, with the assistance of two outer primers (F3 and B3), two inner primers (FIP and BIP), two loop primers (LF and LB), and 1× WarmStart LAMP Master Mix, target sequences are amplified. Then, we acquired end-point fluorescence images of the droplets and calculated the number of droplets with fluorescent signals to obtain the quantitative results of the target (within 1 min). In a positive assay, if the droplet contained at least one target sequence, the droplet yielded a green fluorescent signal. The number of droplets with green fluorescent signals was positively related to the concentration of the samples. In a negative assay, no signals were detected in any of the droplets. Throughout the whole assay, SCADL requires only portable devices (shaking heat block, fluorescence microscope and injection pump) rather than sophisticated instruments, allowing on-site testing of pathogens.

**Fig. 2:**
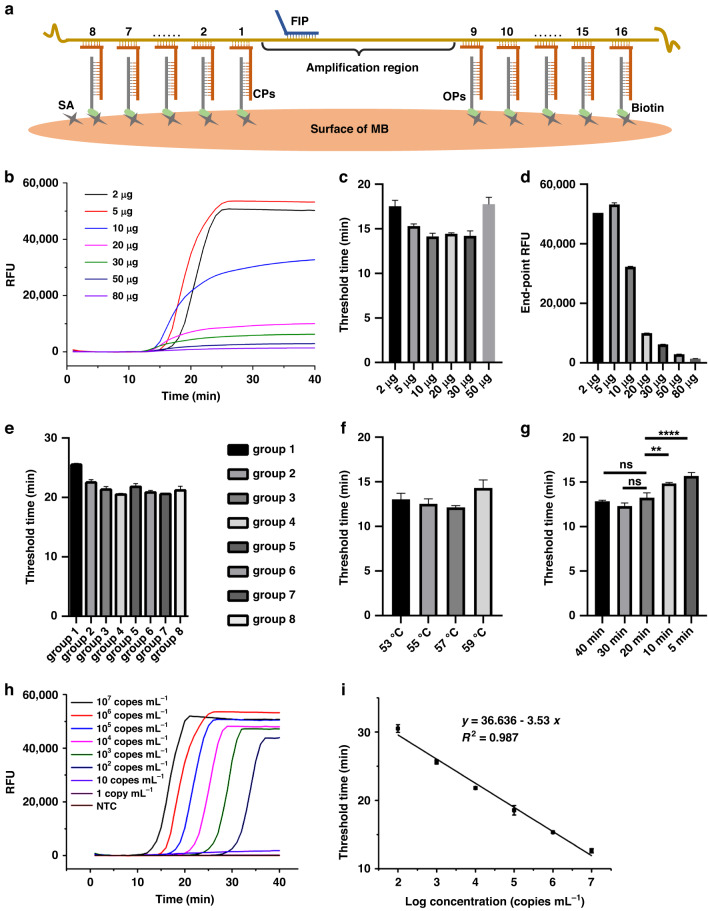
Optimization of conditions for SCADL. **a** Illustration of the regions on the sequence of the N gene with which CPs can hybridize. **b** Real-time fluorescence curves for in vitro transcription (IVT) samples (10^6^ copies per test) with weights of MBs from 2 micrograms to 80 micrograms for capture. Comparison of threshold times (**c**) and end-point fluorescence intensity (**d**) when the amount of MBs used for capture varied from 2 micrograms to 80 micrograms. **e** Comparison of threshold times when using different groups of probes in testing IVD samples (10^4^ copies per test). **f** Comparison of threshold times when the temperature for lysis and capture varied from 53 °C to 59 °C (IVT samples: 10^7^ copies per test). **g** Comparison of threshold times when the time for lysis and capture varied from 5 to 40 min (IVT samples: 10^7^ copies per test). **h** Real-time fluorescence curves for IVT samples (from 1 copy mL^−1^ to 10^7^ copies mL^−1^) tested under the optimal conditions. **i** Linear relationship between the concentrations of IVT samples and the threshold times. All error bars were obtained by calculating the results of three independent experiments. Asterisks represent the significant differences in threshold time between two different groups of conditions (ns, not significant, *p < 0.05, **p < 0.01, ***p < 0.001, ****p < 0.0001)

We optimized the conditions in the SCADL assay, including the amounts of MBs used for capture, as well as the capture probes, time and temperature for lysis and capture. Real-time quantitative RT-LAMP (qRT-LAMP) was conducted to determine the optimal conditions. Conditions with the shortest threshold time are optimal. The CPs lie near but do not cover the amplification region, and each CP was designed with a melting temperature (Tm) between 57–60 °C. We designed 16 separate CPs (8 CPs in front of the amplification region and 8 CPs behind) and divided them into 8 groups (Fig. 2a, SI Tables 2, 3). Sufficient CPs improve the capture efficiency through the base-stacking effect, whereas excessive CPs compete with the limited binding sites on the surface of MBs and affect the enrichment of target sequences on MBs. In the optimization of probes, CPs of Group 4 have the shortest threshold time, which is the optimal condition for capture (Fig. 2e). The amount of MBs is crucial for effective and efficient capture of target sequences, but excessive MBs affect LAMP amplification and fluorescence intensity. The threshold time significantly increased when the weight of MBs was over 30 micrograms (Fig. 2b, c), while the fluorescence intensity decreased when the weight of MBs was over 5 micrograms (Fig. 2b, d). Thus, the optimal amount of MBs for capture and amplification is 5 micrograms. The optimal temperature for capture and lysis is 57 °C (Fig. 2f). Specific capture of target RNA proceeded simultaneously with lysis, without costing extra time. Allowing 20 min or longer for capture and lysis produced significantly better than allowing 10 min (Fig. 2g). Thus, we chose 5 micrograms of MBs, CPs of Group 4, 57 °C, and 20 min for capture in SCADL of SARS-CoV-2. Under the optimal conditions, we detected a group of samples with concentrations from 1 copy mL^−1^ to 10^7^ copies mL^−1^. The limit of detection (LOD) was 100 copies mL^−1^, with a linear relationship between the threshold time and concentration of the targets (*R*^2^ = 0.987) (Fig. 2h, i).

**Fig. 3:**
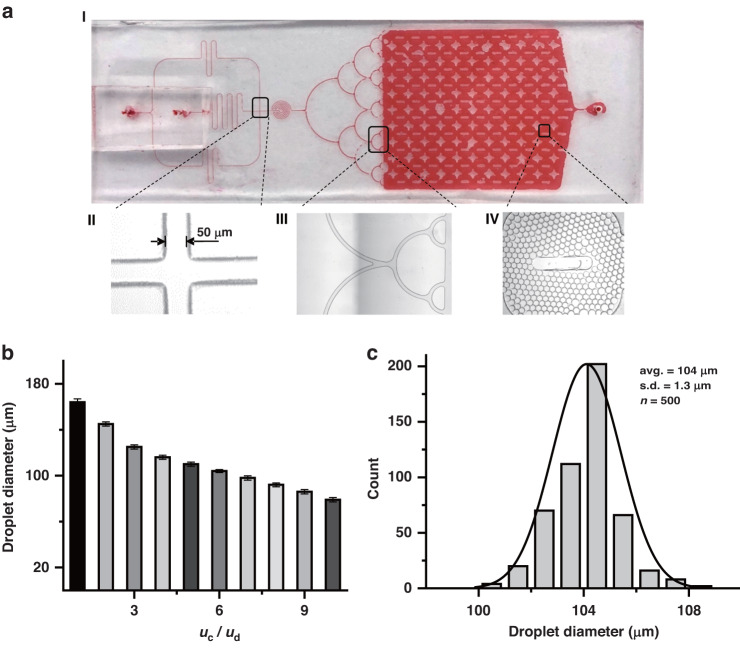
Generation of droplets on microfluidic chips. **a** Structure of the chip (I). It contains three main structures: a crossing channel for the generation of droplets (II), a semicircular channel for dispersion of droplets (III), and a chamber for the collection of droplets (IV). **b** Diameters of the droplets generated under different flow rate ratios. **c** Size distribution of droplets generated under the chosen conditions

The microfluidic chip contains three main structures: a crossing channel for the generation of droplets, a semicircular channel for the dispersion of generated droplets, and a chamber for the collection of droplets (Fig. 3a I). Regarding the crossing channel, one channel is for the mixture of LAMP, and two channels are for the oil phase (Fig. S1a). The width of the channels and the flow rate ratio (*u*_*c*_/*u*_*d*_) are related to the size of the generated droplets, in which *u*_*c*_ is the flow rate of the continuous phase (oil phase) and *u*_*d*_ is the flow rate of the dispersed phase (water phase) (SI Table 4). The sensitivity of the assay is positively associated with droplet size. In the Comsol simulation, the size of the droplet decreased with decreasing channel width (SI Fig. S1b), but the cost for fabrication increased. Thus, we chose to fabricate a channel with a width of 50 μm (Fig. 3a II). The flow rate ratio (*u*_*c*_/*u*_*d*_) is negatively associated with the size of the droplet and positively associated with the frequency of droplet generation and the distance between droplets (Fig. 3b, Fig. S1c–e). The diameter of droplets can be decreased by increasing the flow rate ratio, but this will result in increased consumption of the oil phase. When the flow rate ratio is lower than 6, the droplet diameter is greater than 100 μm, and the number of droplets is lower than 2 × 10^4^ (Fig. 3b). To ensure the generation of sufficient droplets for sensitive SCADL, we chose a flow rate ratio of 6 as the optimal condition and analyzed the size of the generated droplets by counting 500 droplets. More than 2 × 10^4^ droplets were generated, and the average diameter was 104 μm (Fig. 3c). In addition, we designed a semicircular channel to disperse the generated droplets, in which the flow rate is low enough to protect the shape and structure of the droplets (Fig. 3a III, Fig. S1f). The structure of the collection chamber is specifically designed to facilitate constructing a monolayer droplet array for fluorescent signal imaging (Fig. 3a IV). After amplification, we acquired end-point fluorescence images of all the droplets and calculated the number of fluorescent droplets within 1 min.

**Fig. 4:**
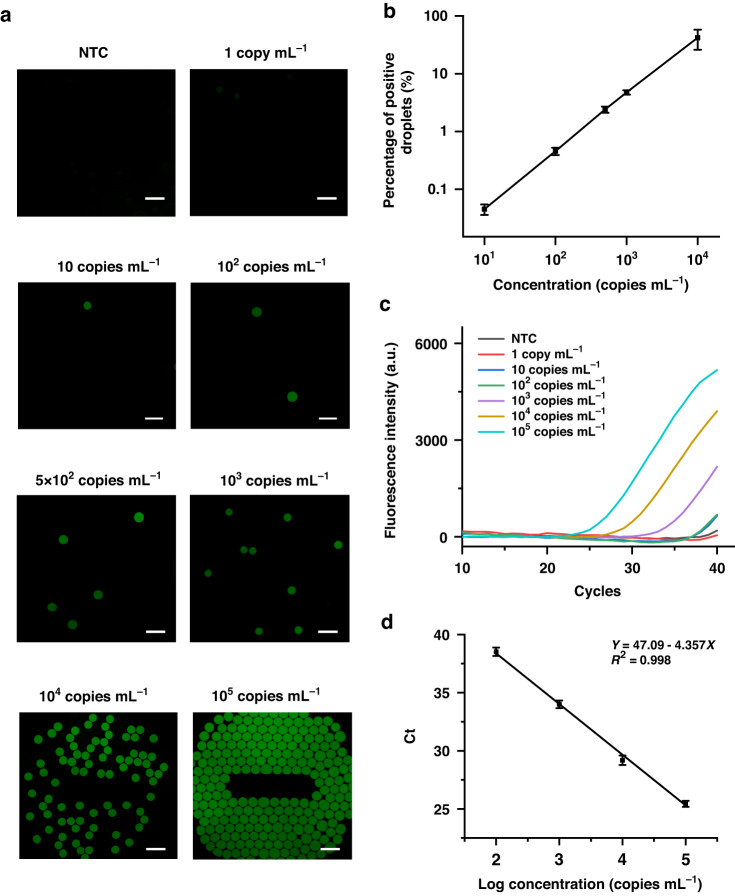
Quantification capability of SCADL for detection of the N gene. **a** End-point fluorescence images of IVT samples with various concentrations (from 1 copy mL^−1^ to 10^5^ copies mL^−1^) (Scale bar: 200 μm). **b** Statistics of positive droplets in the results of end-point fluorescence images. **c** Real-time fluorescence curves for IVT samples (from 1 copy mL^−1^ to 10^5^ copies mL^−1^) tested by RT‒PCR. **d** Linear relationship between the concentrations of IVT samples and the Ct values

To investigate the quantification capability of SCADL, we performed detection of IVT samples with concentrations from 1 copy mL^−1^ to 10^5^ copies mL^−1^. All the samples with concentrations higher than 10 copies mL^−1^ yielded fluorescent signals in the droplets (Fig. 4a). The percentage of positive droplets is precisely correlated to the actual concentration of samples (from 10 copies mL^−1^ to 10^4^ copies mL^−1^) with an excellent linear relationship (Fig. 4b). Once the copy number of target sequences exceeds the number of droplets (2 × 10^4^), the results will be out of the linear range. We can dilute the samples to obtain exact quantitative results. We compared the sensitivity of SCADL with RT‒PCR by conducting a test of samples with concentrations from 1 copy mL^−1^ to 10^5^ copies mL^−1^. The Ct values for samples with concentrations from 100 copies mL^−1^ to 10^5^ copies mL^−1^ range from 38.78 to 25.44, indicating that the LOD in RT‒PCR is 100 copies mL^−1^ (Fig. 4c, d), while the LOD in SCADL is 10 copies mL^−1^. The sensitivity of SCADL is 10-fold higher than that of RT‒PCR.

**Fig. 5:**
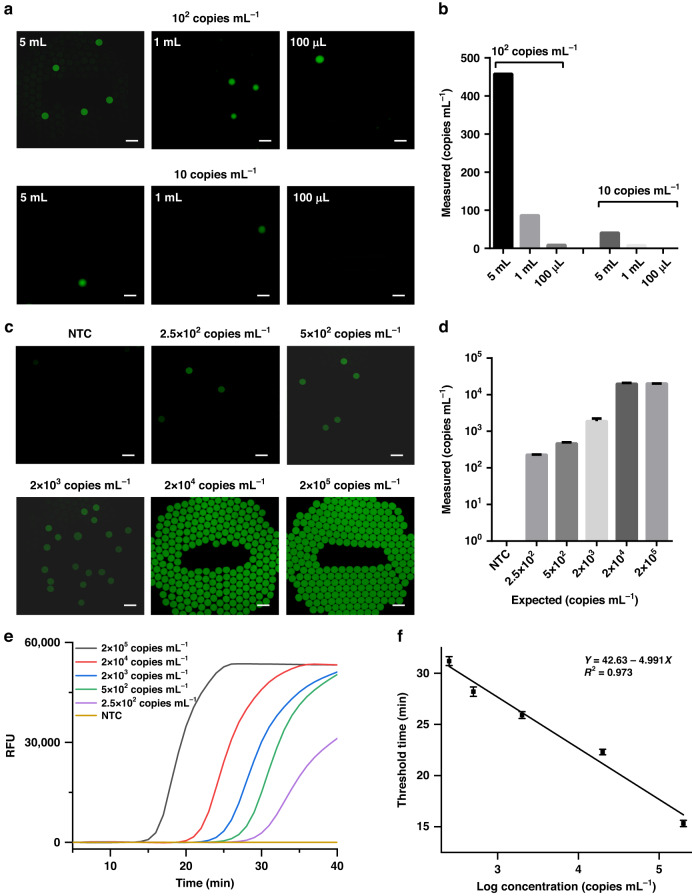
Capacity of SCADL to handle samples with different volumes and assays of SARS-CoV-2. **a** End-point fluorescence images of samples with various volumes (10 copies mL^−1^ and 100 copies mL^−1^) (Scale bar: 200 μm). **b** Measured concentrations of samples with volumes ranging from 100 μL to 5 mL. **c** End-point fluorescence images of commercial standard samples with RNA concentrations ranging from 2.5 × 10^2^ to 2 × 10^5^ copies mL^−1^ (Scale bar: 200 μm). **d** Measured concentrations of the standard samples. **e** Real-time fluorescence curves for standard samples tested by RT-LAMP. **f** Linear relationship between the concentrations of standard samples and the threshold times

To further assess the performance of SCADL in handling samples with different volumes, we measured IVT samples (with concentrations of 10 copies mL^−1^ and 100 copies mL^−1^) with volumes from 100 μL to 5 mL. All the samples with RNA more than 10 copies per test generated positive droplets, whereas other samples generated only negative droplets, regardless of the volume tested (100 μL or 5 mL) (Fig. 5a). The measured concentrations corresponded to the copy number of the targets (Fig. 5b). This indicates that SCADL can detect samples with volumes of up to 5 mL and even larger, while the conventional nucleic acid extraction method can only extract RNA from 100–200 μL of sample^11,26^. We further evaluated the performance of SCADL in the SARS-CoV-2 assay. Due to the lack of clinical samples, we detected commercial standard samples with RNA concentrations from 2.5 × 10^2^ to 2 × 10^5^ copies mL^−1^ and NTC and compared the results with RT-LAMP. All the standard samples of SARS-CoV-2 detected with SCADL yielded positive results, that is, fluorescent droplets in the end-point images (Fig. 5c). The measured concentrations correspond to the actual concentrations of standard samples when they are lower than 2 × 10^4^ copies mL^−1^ (Fig. 5d). For samples with concentrations higher than 2 × 10^4^ copies mL^−1^, such as 2 × 10^5^ copies mL^−1^, because the copy numbers of RNA in samples are beyond the number of droplets, it is difficult to directly acquire accurate quantitative results unless diluted samples are detected. Additionally, all the results detected by SCADL are consistent with the results of RT-LAMP (Fig. 5e, f).

**Fig. 6:**
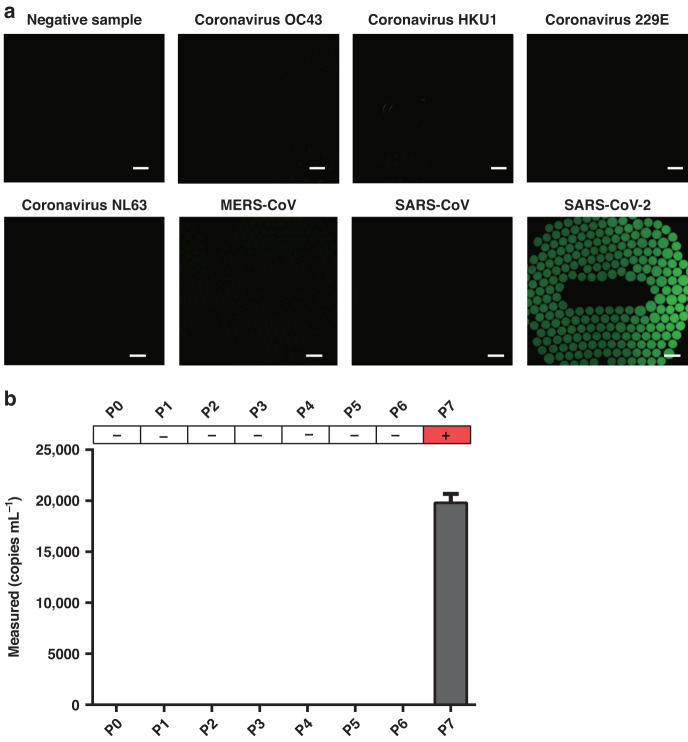
Specificity of SCADL in the SARS-CoV-2 assay. **a** Endpoint fluorescence images of 6 nonspecific samples, 1 sample from a healthy volunteer and 1 positive standard sample (scale bar: 200 μm). **b** Measured concentrations of all the above samples. (P0: Negative sample, P1: Coronavirus OC43, P2: coronavirus HKU1, P3: coronavirus 229E, P4: coronavirus NL63, P5: MERS-CoV, P6: SARS-CoV, P7: SARS-CoV-2)

To investigate the specificity of SCADL, we tested for four kinds of human coronaviruses (coronavirus OC43, coronavirus HKU1, coronavirus 229E and coronavirus NL63), MERS-CoV, SARS-CoV and SARS-CoV-2 in commercial standard samples and 1 swab sample from a healthy volunteer. In three replicate assays, only samples of SARS-CoV-2 yielded positive results (droplets with fluorescent signals) (Fig. 6a, b). The other four kinds of human coronavirus, MERS-CoV, SARS-CoV samples and the sample of healthy volunteers did not cause cross-reaction in the assay (Table 1). This indicates that SCADL is specific enough for the assay of SARS-CoV-2. In the lysis and capture step, only target RNA can be captured by specific CPs and anchored to the surface of MBs, whereas in conventional nucleic acid extraction, total RNA is extracted. Target RNA is bound to MBs, not in solution, decreasing the possibility of aerosol contamination. Therefore, SCADL exhibits higher specificity than other amplification methods based on RNA extraction.

**Table 1:** Specificity of SCADL for SARS-CoV-2 by testing commercial standard samples and samples from healthy volunteers

Number	Virus	Results	Ratio of positive replicates
P0	Non	N	0/3
P1	Coronavirus OC43	N	0/3
P2	Coronavirus HKU1	N	0/3
P3	Coronavirus 229E	N	0/3
P4	Coronavirus NL63	N	0/3
P5	MERS-CoV	N	0/3
P6	SARS-CoV	N	0/3
P7	SARS-CoV-2	P	3/3

## Discussion

We have previously developed a sandwich hybridization-based LAMP for mass screening for malaria, in which RNA targets are captured by a series of specific, tailed probes to remove impurities and nonspecific sequences^27^. Inspired by this successful strategy, we sought to develop ultrasensitive and specific quantification technology at the single-molecule level. In SCADL, each single molecule of the RNA target can be captured and enriched by CP- and OP-modified MBs through an incubation of 20 min without pretreatment or extraction procedures. All the enriched molecules are subsequently dispersed into more than 2 × 10^4^ droplets, in which fluorescent signals of each droplet can be detected and recorded. More importantly, only the target RNA can be captured through hybridization between the target sequences and the CPs, as well as the “tail” of CPs and OPs on MBs, assuring sufficient domains for efficient capture with only 5 μg of MBs and complete enrichment of targets from up to 5 mL of samples by magnetic separation. The specific capture of RNA targets and enrichment based on MBs enable SCADL to perform ultrasensitive and single-molecule assays. Furthermore, capture at the single-molecule level before amplification prevents loss of the RNA target, which is inevitable in strategies based on nucleic acid extraction. In the detection of the N gene of SARS-CoV-2, an LOD of 30 copies μL^−1^ was achieved by traditional LAMP^28^. To improve the sensitivity, amino-modified silicon film was used to extract and enrich nucleic acids, obtaining an LOD of 1 copy μL^−1^^29^. To further realize the absolute quantification of *L. monocytogenes* DNA, digital nucleic acid detection based on microfluidic technology was developed, with an LOD of 10 copies μL^−1^^30^. SCADL combines the advantages of enrichment and droplet digital amplification and thus can realize absolute quantification with a precision (LOD of 10 copies mL^−1^) higher than that of traditional digital amplification strategies.

The second key advance of SCADL is its high specificity. In most of the strategies for nucleic acid assays, the total DNA and RNA are extracted and mixed in the amplification mixture, in which custom designed primers and probes guarantee specificity. However, false-positive results are still a problem waiting for a solution. In SCADL, all the nonspecific sequences and impurities are removed in the washing step after capture. Capture based on specific CPs and LAMP primers provides double insurance for specificity. In addition, all the captured targets are anchored on solid MBs, not in the liquid phase, preventing aerosol contamination of the samples.

Since we integrate droplet generation, amplification, and recording of results in a microfluidic chip, SCADL greatly reduces the need for expert personnel and precision instruments. In other droplet amplification strategies based on microfluidic chips, droplets are generated on chips and finally collected outside the chip^31^. The recording and analysis of the results usually rely on sophisticated instruments, making the detection only carried out in well-equipped laboratories. In contrast, SCADL can be widely used in regions lacking precise instruments and professionals. We can further realize a fully automatic sample-in-result-out assay by integrating the function of capture and lysis on a platform based on the SCADL strategy. In that case, the platform is capable of handling samples of infectious diseases with high transmissibility rates in common laboratories. In future work, we will commit to realizing multiplex assays, improving throughput, and developing automatic assay platforms.

## Conclusions

In summary, we developed single-molecule RNA capture-assisted digital RT-LAMP by using a microfluidic chip to realize droplet generation, amplification, and analysis of results. We validated the performance of SCADL by detecting the N gene of SARS-CoV-2, and the results showed the ultrasensitivity, specificity, quantifiability, rapidity and user-friendliness of the approach and its ability to greatly reduce the dependence on expensive equipment and skilled personnel. We anticipate that the SCADL strategy can be widely adopted in the control and prevention of infectious diseases, as well as quantitative assays in biomedical research and clinical diagnosis.

### Supplementary information

Supplemental Material
