# Supplementary material for: Single-molecule RNA capture-assisted droplet digital loop-mediated isothermal amplification for ultrasensitive and rapid detection of infectious pathogens
Source: Microsyst Nanoeng. 2023 Sep 25;9:118. doi: 10.1038/s41378-023-00576-2 (PMC10519972; doi:10.1038/s41378-023-00576-2)
Supplement: Supplementary file 1 — Supplemental Material [file 41378_2023_576_MOESM1_ESM.docx]

Single-molecule RNA capture assisted droplet digital LAMP for ultrasensitive and rapid detection of infectious pathogens

Liying Jiang^1,5^, Xianghao Lan^1^, Linjiao Ren^1^, Zhiyuan Jin^2^, Xuchen Shan^4,*^, Mingzhu Yang^3,*^, and Lingqian Chang^2,*^

*^1^ School of Electrical and Information Engineering, Zhengzhou University of Light Industry, Zhengzhou 450002, China.*

*^2^ Key Laboratory of Biomechanics and Mechanobiology of Ministry of Education Beijing Advanced Innovation Center for Biomedical Engineering, School of Biological Science and Medical Engineering, Beihang University, Beijing 100191, China.*

*^3^ Beijing Research Institute of Mechanical Equipment, Beijing 100143, China*

*^4^ School of Physics, Beihang University, Beijing, 100191 China*

*^5^ Academy for Quantum Science and Technology, Zhengzhou University of Light Industry, Zhengzhou 450002, China.*

** Correspondence: Mingzhu Yang (yixin051@163.com), Xuchen Shan (shanxuchen@buaa.edu.cn), and Lingqian Chang (lingqianchang@buaa.edu.cn)*

**Materials and methods**

**Materials**

Chemical reagents, including sodium chloride (NaCl), EDTA, Triton X-100, Tris-HCl and BSA were obtained from Sigma-Aldrich (Shanghai, China). Polydimethylsiloxane (PDMS) was purchased from Dow Corning (Midland, USA). Phosphate buffered brine (PBS,10×), and ethanol were from Sangon Biotech (Shanghai, China). Isopropanol, SU8 negative photoresist and PM-THINNER developer were obtained from Suzhou Research Semiconductor Co., Ltd. (Suzhou, China). Streptavidin modified magnetic beads (Dynabeads MyOne Streptavidin C1) were purchased from Invitrogen (USA). 2× WarmStart LAMP Kit (E1700S) were purchased from New England Biolabs (Beijing, China). All the primers and probes were synthesized by Sangon (Shanghai, China). Proteinase K and Nuclease-free water were obtained from Tiangen Biotech (Beijing, China). Lysis buffer and washing buffer were laboratory-prepared.

Commercial standard samples were from Guangzhou BDS Biological Technology Co., Ltd (Guangzhou, China). In vitro transcribed (IVT) RNA was transcribed from plasmid inserted with the sequences of N gene by using MEGAscript T7 transcription kit (Thermo Fisher Scientific, USA) and quantified by using Nanodrop with an absorbance at 260 nm.

**Instrument**

Bio-rad T100 gradient PCR instrument was obtained from Bio-rad Company (California, USA). Shaking heat-block thermomixer was purchased from Eppendorf (Hamburg, Germany). Electric constant temperature blast drying box was purchased from Shanghai Xinmiao medical equipment manufacturing Co., Ltd. (Shanghai, China). URE-2000/17 UV depth lithography machine was purchased from Shenyang Scientific Instruments Co., Ltd. (Shenyang, China). YZD08-2C plasma cleaning machine was purchased from Saiot (Beijing) Technology Co., Ltd. (Beijing, China). XFP01-BD (2) injection pump was purchased from Suzhou Xunfei Scientific Instruments Co., Ltd. (Suzhou, China). NEXCOPE inverted fluorescence Biomicroscope NIB900 was purchased from Chongqing Liuhui Technology Co., Ltd. (Chongqing, China).

**Methods**

**Design and fabrication of the chips**

We used the Flow-focusing structure as the droplet generation structure, and set the channel width to 50 μm. We used a spiral structure with a channel width of 100 μm to stabilize the droplets, and the droplet splitting structure with a channel width of 100 μm to made the droplets more evenly dispersed in the collection chamber. We designed the collection chamber of 2.23 cm in length and 2.09 cm in width, and set up a support to prevent the collection chamber from collapsing due to its excessive area. We used Auto CAD software to draw the chip structure and make a photolithographic mask version (Fig. S2). We used SU-8 lithography process to process the master mode of droplet microfluidic chip. Firstly, we cleaned the silicon wafer and used the spin coating machine to coat the silicon wafer with a layer of 50 μm thick SU-8 2050. After a two-step baking process (65 ^o^C for 3 minutes and 95 ^o^C for 8 minutes), the samples were exposed to ultraviolet light for 13 seconds through a photolithographic mask. Then, the two-step post-exposure baking process (65 ^o^C for 2 minutes and 95 ^o^C for 6 minutes) was carried out. The processing and preparation of the master mold was completed after clean the residual photoresist with acetone and isopropanol. The polydimethylsiloxane (PDMS) and the curing agent were mixed at 10:1 by weight, degassed in a vacuum dryer and poured into the SU-8 pattern mold. The PDMS layer was heated and cured overnight at 80 ^o^C and bonded to each other, and the opposite microstructure was formed by demoulding process.

Finally, the glass slip was glued to the top of the microfluidic structure layer by oxygen plasma treatment, and was placed in an electrothermal constant temperature blast drying box at 80 ^o^C for 20 min. Finally, the assembly of the microfluidic chip which can generate droplets is completed.

**COMSOL simulation of the chips for droplets generation**

We used the Level-set method in COMSOL Multiphysics to construct models for different flow channel widths and droplet splitting structures for the desired droplet microfluidic chip. In Level-set method, the liquid flow velocity of two-phase flow is low at microscopic size, where the two phases can be viewed as homogeneous incompressible fluid, and the effect of gravity of the fluid on the flow rate and velocity is minimal and negligible under the conditions of micrometer generated droplets. In order to study the dynamics of droplet deformation, the Navier-Stokes equation needs to be solved numerically to obtain the simplified N-S equation as below.

$$\nabla\cdot\boldsymbol{u}\text{ }\text{=0}$$

$$\frac{\rho\partial\text{u}}{\partial t}+\rho\nabla\cdot\left( \boldsymbol{uu} \right)=-\nabla p+\nabla\cdot\left[ \mu\left( \nabla\boldsymbol{u}\text{ }\text{+}\nabla\boldsymbol{u}^{\text{T}} \right) \right]+\boldsymbol{F}$$

$$\frac{\partial\emptyset}{\partial t}+\boldsymbol{u}\boldsymbol{\cdot}\nabla\emptyset=\gamma\nabla\cdot[\varepsilon\nabla\emptyset-\emptyset\left( 1-\emptyset\right)\frac{\nabla\emptyset}{|\nabla\emptyset|}]$$

***u*** is the fluid velocity, *p* is the fluid pressure, *ρ* is the dynamic density of the fluid, *μ* is the dynamic viscosity coefficient, ***F*** is the surface tension, $\emptyset$ denotes the level set function, and *γ* and *ε* are numerically stabilized parameters. Where *ρ* and *μ* can be determined by the following equations:

$$\rho=\rho_{c}+\left( \rho_{c}-\rho_{d} \right)\emptyset$$

$$\mu=\mu_{c}+\left( \mu_{c}-\mu_{d} \right)\emptyset$$

where *ρ_c_, ρ_d_, μ_c_, μ_d_* denote the density and viscosity of the continuous and dispersed phases, respectively, and the effective diameter of microdroplets *d_eff_*  is calculated as follows:

$$d_{eff}=2\boldsymbol{\cdot}\sqrt[\boldsymbol{3}]{\frac{3}{4\pi}\int\left( \emptyset>0.5 \right)d\Omega}$$

We set the channel width of the droplet formation structure in COMSOL as 40~70 μm, the continuous phase flow rate as 0.005 m s^-1^ and the dispersed phase velocity as 0.005 m s^-1^ to explore the relationship between the width of different channels and the droplets generated. Then we set the width of the channel as 50 μm, the velocity of the dispersed phase (*u*_d_) as 0.005 m s^-1^, and simulate the velocity of the continuous phase (*u*_c_) as 0.005~0.07 m s^-1^, and the velocity ratio of the continuous phase to the dispersed phase was 1: 14, to explore the relationship between different velocity ratio and droplet diameter and frequency. At the same time, we built a droplet splitting structure to explore whether the velocity of each outlet of the droplet splitting structure was consistent.

**Preparation of oligonucleotide probes conjugated MBs**

100 μL of streptavidin modified MBs (10 mg mL^-1^) were placed in a centrifuge tube and placed on a magnetic force rack. After the supernatant was removed, 2× binding and washing buffers were used to rinse once (Binding and Washing (B&W) Buffer (2×): 2 M NaCl, 1 nM EDTA, 10 mM Tris-HCl (pH 7.5). After removing the supernatant, we added 200 μL B&W buffer to resuspend, and then added 20 μL 100 μmol L^-1^ OPs and 180 μL water to mix evenly. Subsequently, we oscillated the mixture at room temperature for 30 minutes, and placed the magnetic beads of coupled oligonucleotides on the magnetic rack after the oscillation was completed. We washed it with 1× binding buffer for 3 times, removed the unbound OPs, and then washed the supernatant for the last time. Finally, we resuscitated it with 1 mL nuclease-free water (1 ml 0.1% BSA) and stored it at 4 ^o^C.

**Procedures for** **RT-LAMP**

Lysis mixture (1 mL) containing 1 nM of CPs, 0.92 g L^-1^ PK, 1×lysis buffer, 5 μL 1 mg mL^-1^ OPs conjugated MBs and appropriate amount of sample (IVT samples or standard samples) were mixed and incubated at 57 ^o^C for 20 minutes (1500 rpm) in a shaking heat-block thermomixer (Eppendorf). After capture, MBs were washed 2 times with 1× washing buffer. Then 25 μL LAMP mixture was added to complete RT-LAMP. In real-time quantitative RT-LAMP, RT-LAMP mixture containing 12.5 μL 2× WarmStart LAMP Master Mix, 0.5 μL 50× LAMP Fluorescent Dye, 2.5 μL 10× primers, 9.5 μL nuclease-free water and washed MBs were incubated for 40 minutes at 65 ^o^C to complete amplification. RT-LAMP reaction was conducted in Biorad CFX96 at 65 ^o^C for 40 minutes, with 1-minute intervals to record fluorescence intensity. 10× primer mix including a pair of outer primers (F3 and B3, 2 μM), a pair of inner primers (FIP and BIP, 16 μM) and a pair of loop primers (LF and LB, 8 μM). The sequences of all the primers are listed in table 1.^1^

**Optimization of conditions for capture**

In the optimization of the amount of MBs on RT-LAMP, we added 2 μL, 5 μL, 10 μL, 20 μL, 30 μL, 50 μL, and 80 μL of 1 mg mL^-1^ OPs conjugated MBs into tubes, placed the tubes on the magnetic rack and removed the supernatant. 1× capture probe of group 4, 57 ^o^C, and 20 minutes were used for capture, and 1μL 10^6^ copies μL^-1^ IVT sample were detected. In optimization of capture probes, 1× capture probe of group 1, group 2, group 3, group 4, group 5, group 6, group 7, and group 8 were used for capture (table 2 and table 3). And in the optimization of temperature for capture and lysis, the procedure was conducted at 53 ^o^C, 55 ^o^C, 57 ^o^C, and 59 ^o^C, respectively, while in optimization of capture time, we incubated the lysis and capture mixture for 5, 10, 20, 30, 40 minutes.

**Procedure of Digital-LAMP on the system**

The droplet generation chip and droplet formation device were first integrated with the microfluidic chip (Fig. S3), and the prepared surfactant premixed mineral oil and water reagent were injected into the microfluidic chip from the continuous phase inlet and the dispersed phase inlet, respectively. The freshly prepared surfactant premixed mineral oil was filled within the whole chip before the test and placed under a fluorescence microscope for observation. The velocity of the dispersed phase (*u*_d_) was subsequently fixed at 1 μL min^-1^, and the velocity of the continuous phase (*u*_c_) was set from 1 to 10 μL min^-1^, respectively. We observed the diameter size of droplets in the collection chamber under a fluorescence microscope.

In accordance with the above steps, the mineral oil premixed with surfactant and LAMP mixture, containing MBs with captured target sequence, were connected to the continuous phase inlet and the dispersive phase inlet of the droplet microfluidic chip, respectively. Subsequently, the velocity of the continuous phase (*u*_c_) was set to 6 μL min^-1^ and the velocity of the dispersed phase (*u*_d_) was set to 1 μL min^-1^. Than we collected generated droplets in the collection chamber, and incubated the collected droplets with a water bath heater at 57^o^C for 20 minutes. Then we placed the chip under a fluorescence microscope for observation and analysis of the number of positive droplets after the amplification was completed.

**Assessment of sensitivity**

Following the previous procedure for MBs capture of enriched target sequences, NTC and samples with concentration from 1 copies mL^-1^~10^7^copies mL^-1^ were detected. Lysis mixture was added to each tube. Lysis mixture (1 mL) containing 1 nM of CPs, 0.92 g L^-1^ PK, 1×lysis buffer, 1 μL 1 mg mL^-1^ OPs conjugated MBs and appropriate amount of sample (NTC and IVT samples with concentration of 1copies mL^-1^~10^7^copies mL^-1^) were mixed and incubated at 57 ^o^C for 20 minutes (1500 rpm) in a shaking heat-block thermomixer. After capture, MBs were washed 2 times with 1× washing buffer. 10 μL LAMP mixture was added to each tube. The droplets were then generated using a droplet generation platform, in which we set velocity of 6 μL min^-1^ for the continuous phase and 1 μL min^-1^ for the dispersed phase. Then the collected droplets were incubated in a water bath at 64^o^C for 20 minutes. Finally, we observed the number of positive droplets and completed statistical analysis.

**Assessment of specificity**

We conducted the detection of negative samples and coronavirus OC43, coronavirus HKU1, coronavirus 229e, coronavirus NL63, MERS-CoV, SARS-Cov and SARS-CoV-2 with a concentration of 10^6^ copies mL^-1^. After capture, MBs were washed 2 times with 1× washing buffer. Then added 10 μL of LAMP mixture. The LAMP mixture was then generated into droplets and collected using a droplet generation platform. The collected droplets were incubated in a water bath at 64^o^C for 20 minutes to complete LAMP amplification, and then placed under a fluorescent microscope to observe the presence of positive droplets.

**Detection of standard sample**

We detected commercial standard samples with RNA concentrations from 2.5 × 10^2^ to 2 × 10^5^ copies mL^-1^ and NTC samples, followed the procedure of Digital-LAMP testing IVT samples, and finally observed the number of positive droplets and analyzed the end-point fluorescent images.


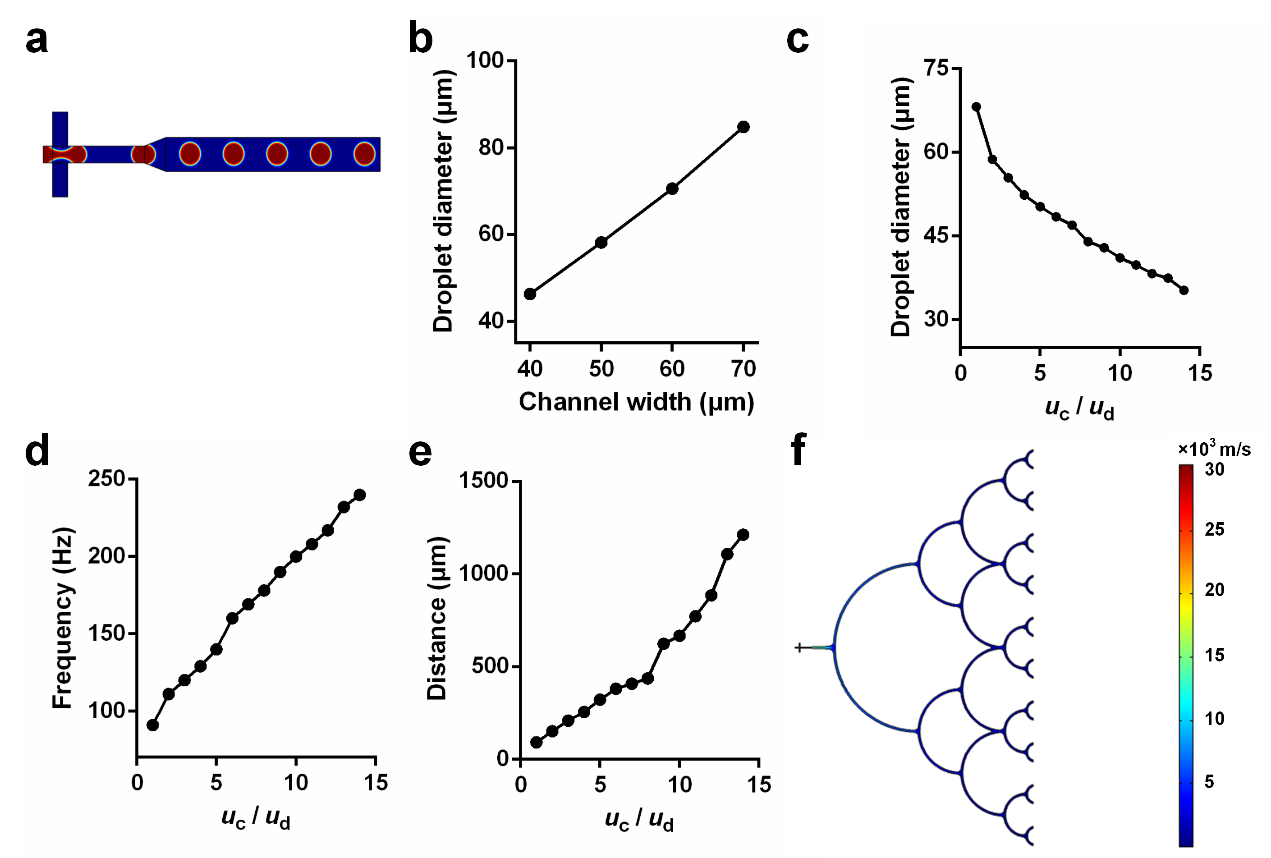


**Figure S1.** (a) Schematic diagram of generating droplet structure model in COMSOL. (b) Relationship between width of channel and size of droplet. (c) Relationship between two-phase different flow rate ratio and diameter of droplet. (d) Relationship between two-phase different flow rate ratio and frequency of generating droplet. (e) Relationship between two-phase different flow rate ratio and distance between droplets. (f) Droplet splitting structure model in COMSOL.


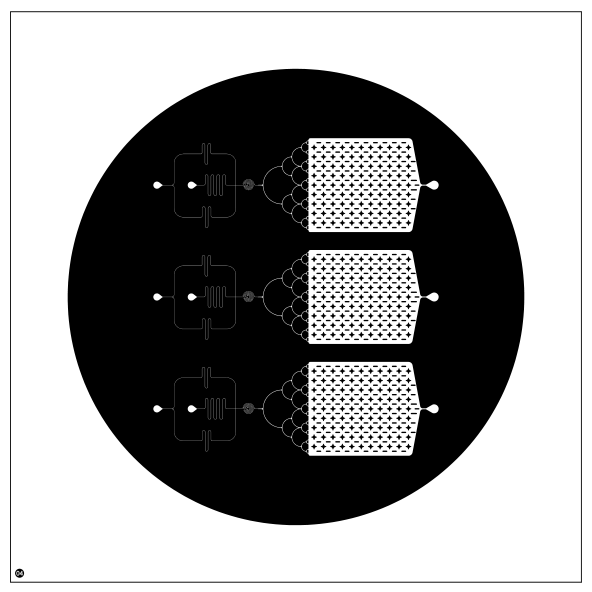


**Figure S2. Photolithographic mask pattern for processing droplet microfluidic chips.**


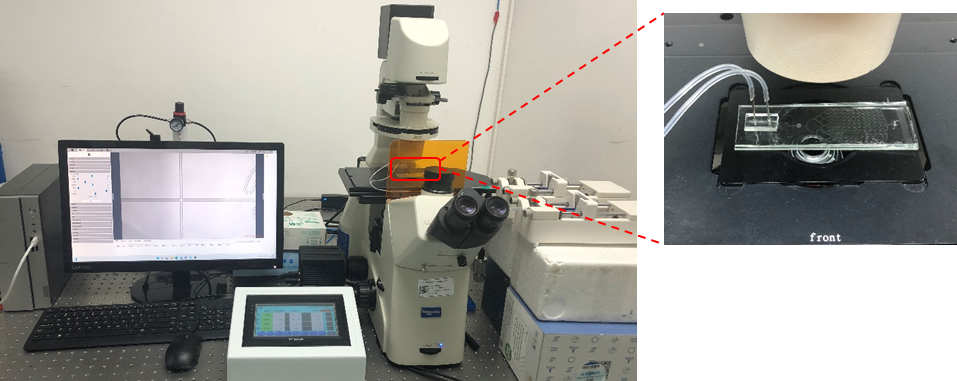


**Figure S3. Droplet generation platform.**


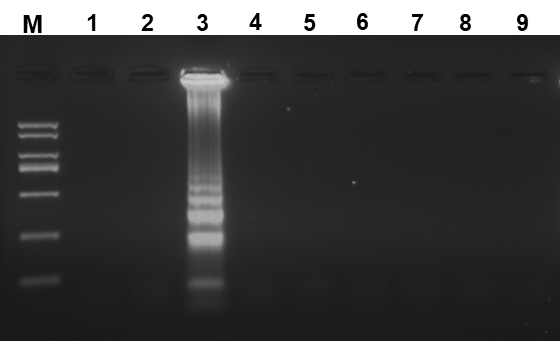


**Figure S4. Gel electrophoresis image of the product after LAMP amplification**: Marker (M), negative samples (1), SARS-Cov (2), SARS-CoV-2 (3), Coronavirus OC43 (4), Coronavirus HKU1 (5), Coronavirus 229e (6), Coronavirus NL63 (7), MERS-CoV (8), MERS-CoV (9).

**Table 1. Primer sequences used for RT-LAMP of N gene in this study.**

| Primer | Sequence (5’ to 3’) |
| --- | --- |
| N-F3 | TGG CTA CTA CCG AAG AGC T |
| N-B3 | TGC AGC ATT GTT AGC AGG AT |
| N-FIP | TCT GGC CCA GTT CCT AGG TAG TCC AGA CGA ATT CGT GGTGG |
| N-BIP | AGA CGG CAT CAT ATG GGT TGC ACG GGT GCC AAT GTG ATCT |
| N-LF | GGA CTG AGA TCT TTC ATT TTA CCG T |
| N-LB | ACT GAG GGA GCC TTG AAT ACA |

**Table 2. Capture probe sequences**

| Probe | Sequences (probe + tail) | Tm (^o^C) |
| --- | --- | --- |
| 1 | GCCAGTTGAATCTGAGGGTCCTTTTTCTGCTAGTATCAGACCGCTGTC | 57.9 |
| 2 | ACTGCGTTCTCCATTCTGGTTACTTTTTTCTGCTAGTATCAGACCGCTGTC | 58.2 |
| 3 | GTTGTTTTGATCGCGCCCCTTTTTCTGCTAGTATCAGACCGCTGTC | 57.7 |
| 4 | GGTAAACCTTGGGGCCGACTTTTTCTGCTAGTATCAGACCGCTGTC | 58.7 |
| 5 | CGGTGAACCAAGACGCAGTATTATTGTTTTTCTGCTAGTATCAGACCGCTGTC | 57.9 |
| 6 | CTTCCTTGCCATGTTGAGTGAGAGTTTTTCTGCTAGTATCAGACCGCTGTC | 57.7 |
| 7 | CCTTGTCCTCGAGGGAATTTAAGGTTTTTTCTGCTAGTATCAGACCGCTGTC | 58.4 |
| 8 | GGACTGCTATTGGTGTTAATTGGAACGTTTTTCTGCTAGTATCAGACCGCTGTC | 58 |
| 9 | GGCAATGTTGTTCCTTGAGGAAGTTTTTTTCTGCTAGTATCAGACCGCTGTC | 58 |
| 10 | CTCCCTTCTGCGTAGAAGCCTTTTTTTTTCTGCTAGTATCAGACCGCTGTC | 59 |
| 11 | GCTTGACTGCCGCCTCTGTTTTTCTGCTAGTATCAGACCGCTGTC | 59.3 |
| 12 | GACTACGTGATGAGGAACGAGAAGAGTTTTTCTGCTAGTATCAGACCGCTGTC | 58.1 |
| 13 | CCTGGAGTTGAATTTCTTGAACTGTTGCTTTTTCTGCTAGTATCAGACCGCTGTC | 58.5 |
| 14 | GGAGAAGTTCCCCTACTGCTGTTTTTCTGCTAGTATCAGACCGCTGTC | 57.1 |
| 15 | CCATTGCCAGCCATTCTAGCATTTTTCTGCTAGTATCAGACCGCTGTC | 58 |
| 16 | AAAGCAAGAGCAGCATCACCGTTTTTCTGCTAGTATCAGACCGCTGTC | 58.4 |

**Table 3. Group of capture probe**

| Group | Probe |
| --- | --- |
| 1 | Probe 1 + Probe 9 |
| 2 | Probe 2 + Probe 1 + Probe 9 + Probe 10 |
| 3 | Probe 3 + Probe 2 + Probe 1 + Probe 9 + Probe 10 + Probe 11 |
| 4 | Probe4 + Probe 3 + Probe 2 + Probe 1 + Probe 9 + Probe 10 + Probe 11 + Probe 12 |
| 5 | Probe 5 + Probe4 + Probe 3 + Probe 2 + Probe 1 + Probe 9 + Probe 10 + Probe 11 + Probe 12 + Probe 13 |
| 6 | Probe 6 + Probe 5 + Probe4 + Probe 3 + Probe 2 + Probe 1 + Probe 9 + Probe 10 + Probe 11 + Probe 12 + Probe 13 + Probe 14 |
| 7 | Probe 7 + Probe 6 + Probe 5 + Probe4 + Probe 3 + Probe 2 + Probe 1 + Probe 9 + Probe 10 + Probe 11 + Probe 12 + Probe 13 + Probe 14 + Probe 15 |
| 8 | Probe 8 + Probe 7 + Probe 6 + Probe 5 + Probe4 + Probe 3 + Probe 2 + Probe 1 + Probe 9 + Probe 10 + Probe 11 + Probe 12 + Probe 13 + Probe 14 + Probe 15 + Probe 16 |

**Table 4. Velocity to velocity ratio of continuous phase (oil phase) and dispersed phase (water phase) in droplet formation**

| *u*_c_ (μL min^-1^) | *u*_d_ (μL min^-1^) | *u*_c_ / *u*_d_ | Error (%) |
| --- | --- | --- | --- |
| 1 | 1 | 1 | 2.873 |
| 2 | 1 | 2 | 1.82 |
| 3 | 1 | 3 | 1.89 |
| 4 | 1 | 4 | 1.75 |
| 5 | 1 | 5 | 1.86 |
| 6 | 1 | 6 | 1.29 |
| 7 | 1 | 7 | 1.89 |
| 8 | 1 | 8 | 1.68 |
| 9 | 1 | 9 | 1.9 |
| 10 | 1 | 10 | 1.91 |

1. Mautner, L. et al. Rapid point-of-care detection of SARS-CoV-2 using reverse transcription loop-mediated isothermal amplification (RT-LAMP). *Virology Journal* **17** (2020).
